# Echocardiographic Determination of Percutaneous Central Venous Catheters in the Superior Vena Cava: A Prospective Cohort Study

**DOI:** 10.3390/children9050624

**Published:** 2022-04-27

**Authors:** Yao-Sheng Wang, Hsin-Chun Huang, Yu-Chen Liu, I-Lun Chen

**Affiliations:** 1 Department of Pediatrics, Chiayi Chang Gung Memorial Hospital and Chang Gung University College of Medicine, Chiayi 613, Taiwan; kvnobulu2@cgmh.org.tw; 2 Department of Pediatrics, Kaohsiung Chang Gung Memorial Hospital, and Chang Gung University College of Medicine, Kaohsiung 833, Taiwan; drhhuang@hotmail.com (H.-C.H.); yugen28@cgmh.org.tw (Y.-C.L.); 3 School of Traditional Chinese Medicine, College of Medicine, Chang Gung University, Linkou 333, Taiwan

**Keywords:** neonate, neonatal intensive care unit, peripherally inserted central catheter, percutaneous central venous catheter, prematurity, ultrasonography

## Abstract

Malposition of percutaneous central venous catheters (PCVCs) in the superior vena cava (SVC) is common. We previously showed that real-time sonography was safer and faster than radiography in identifying PCVC tip location in the inferior vena cava (IVC). However, in preterm infants, determining PCVC tip location in the SVC is complicated by endotracheal or nasogastric tubes in situ and emphysematous lung conditions. We aimed to find an appropriate sonographic view by which to assess PCVC tip location in the SVC compared to the sonographic examination of PCVC in the IVC. Neonates (*n* = 50) with PCVCs in the SVC were enrolled and their data (gestational age, gender, birth weight, body weight at intervention, repositioning rate, and duration of tip assessment) were compared with retrospective data of 50 neonates with PCVCs in the IVC. The mean gestational age in the groups of IVC and SVC was 31.43 weeks and 32.16 weeks, respectively. The mean birth weight in the groups of IVC and SVC was 1642.18 g and 1792.00 g, respectively. Placement of an S12-4 ultrasound sector transducer to obtain clear parasternal views of the aorta allows visualization of PCVC tips in the SVC and near the cavoatrial junction. PCVC repositioning rates were not significantly different between the two groups (*p* = 0.092). Sonography examinations in the SVC had a longer duration than those in the IVC (*p* < 0.001). Sonography provides an accurate method for determining PCVC tip location in the SVC.

## 1. Introduction

Percutaneous central venous catheter (PCVC) insertion is a common procedure performed for critically ill patients, especially on very preterm neonates in the neonatal intensive care unit (NICU), to prepare for fluid infusion, prolonged medication administration, and parenteral nutrition. PCVCs are inserted into peripheral veins on the arms or legs, and finally end up within the superior vena cava (SVC) and near the cavoatrial junction, or the inferior vena cava (IVC) near the right atrium. Among central lines in neonates, PCVCs are associated with more indwelling complications than other central lines (e.g., umbilical arterial catheter or umbilical vein catheter) [1]. The malposition of PCVCs in the SVC may lead to life-threatening complications, including cardiac arrhythmias [2], upper extremity deep vein thrombosis [3], pericardial perforation/effusion/tamponade [4,5], pleural effusion [5], and chylothorax [6]. Although these complications are rare, they may still cause prolonged hospitalization and will need to be replaced or removed. A case report documented that a PCVC line was misplaced into the azygous vein, which was detected only on the second read of fluoroscopic imaging [7]. Therefore, it becomes crucial to confirm the catheter tip position correctly in the SVC to prevent catheter migration or dislodgement. The equations of the inserted length of PCVCs provide us with the optimal insertion length of PCVCs before placement [8]. Conventional radiography with or without contrast enhancement, ultrasonography, and bedside electrocardiograph-guided insertion have been reported as tip detection tools [9,10,11]. However, a PCVC tip that is assessed by conventional radiography with contrast has the risk of radiation and contrast exposure. The advantages of ultrasonography examination are that it provides real-time assessment of the tip position, minimal handling of the neonate, a guide for repositioning of central lines, portability, lack of ionizing radiation, and the absence of known health risks [12]. Besides, patients do not require sedation, and examiners can easily capture both static and cine images that include characterization of flow dynamics with color and spectral Doppler [12]. In our previous experience, the obstacles of endotracheal or nasogastric tubes in situ and emphysematous lung conditions make PCVC insertion in the SVC more difficult to assess by ultrasonography than insertion in the IVC. In our NICU, PCVC tip location in the IVC has been routinely assessed by ultrasonography for over one year and has been published as having a lesser pull-back rate and shorter checking time as compared to radiography [10]. Thus, the purpose of this study was to determine the appropriate view by which to detect the tip location of PCVCs that are placed in the SVC and to compare related data from evaluating PCVCs placed in the IVC.

## 2. Patients and Methods

### 2.1. Study Design and Sample

This prospective cohort study was conducted in a level III, thirty-three-bed NICU from January 2021 to December 2021. Fifty neonates (SVC group) who had PCVCs in the SVC were enrolled to assess the location of the tip by ultrasonography. Because the assessment of tip location of PCVCs in IVC in the NICU had already been routinely performed by ultrasonography in our previous study, the medical records data of fifty neonates who had PCVCs in the IVC (IVC group) were retrospectively reviewed as the comparative data. Patients’ gestational age (GA), birth weight (BW), gender, age, and body weight at the moment of intervention were matched between the SVC and IVC groups. All tip positions of PCVCs were finally confirmed by radiography after ultrasonography. The repositioning rate, duration of intervention, duration of tip assessment by sonography, and the duration of tip assessment by radiography, the complication of PCVCs, such as catheter-related bloodstream infection (CRBSI), occlusion, and unexpected removal, were compared between SVC and IVC groups. The SVC group was further divided into invasive ventilation and non-invasive ventilation subgroups for analyzing the influence of ventilation on finding PCVC tips located in SVC. Invasive ventilation is defined as positive pressure delivered to a patient’s lung via an endotracheal tube, whereas non-invasive ventilation is via a nasal device. The repositioning rate means the rate of repositioned PCVCs after final radiography. The duration of the intervention is defined as the time from inserting the needle through the skin to advancing to the estimated length. The duration of tip assessment by sonography is defined as the time from putting the probe on the chest wall until visualization of the proper tip location, including normal saline fluid flushing, tip location adjustment, and a four-chamber view exam. The duration of tip assessment by radiography is defined as the time from ordering and taking the radiography until the confirmation by the neonatologist. CRBSI is defined according to the Nosocomial Infections Surveillance System from Taiwan Centers for Disease control in 2018. An eligible bloodstream infection (BSI) organism is identified and an eligible central line is present on the date of laboratory-confirmed BSI or the day before. An eligible central line means that the central catheter has been already in place for more than two calendar days. The unit of CRBSI is per 1000 central-venous-catheter days. An unexpected removal was defined as removal that resulted from an unresolvable complication.

### 2.2. Methods

**Figure 1:**
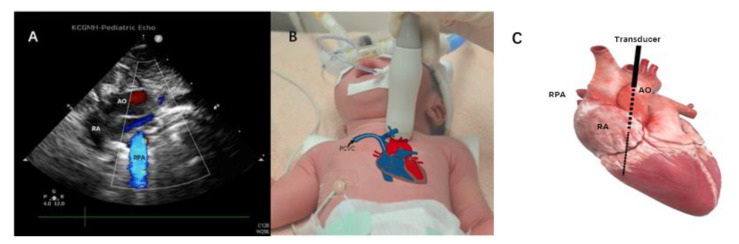
The probe was placed on the baby’s left parasternum, at the upper border between nipples. The black arrow indicates the percutaneous central venous catheter (PCVC) in situ. (**A**) Aorta in the left parasternal longitudinal view, (**B**) location of the transducer, and (**C**) the heart anatomy with the probe position indicates a black solid line and black dash line for the plane of ultrasound. AO, aorta; RPA, right pulmonary artery; RA, right atrium.

**Figure 2:**
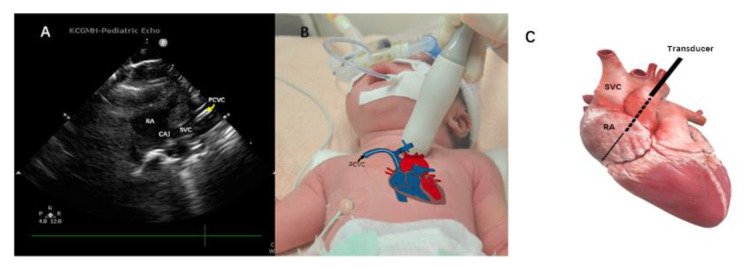
After detecting the aorta, the probe was slightly tilted to the baby’s right side showing (**A**) PCVC, an image of contrasting double-contoured echo structure (yellow curved arrow) in the SVC; (**B**) the location of the transducer; and (**C**) the heart anatomy with the probe position indicated in a black solid line and a black dash line indicating the plane of ultrasound. The black arrow shows PCVC in situ. CAJ, cavoatrial junction; PCVC, percutaneous central venous catheter; RA, right atrium; SVC, superior vena cava.

**Figure 3:**
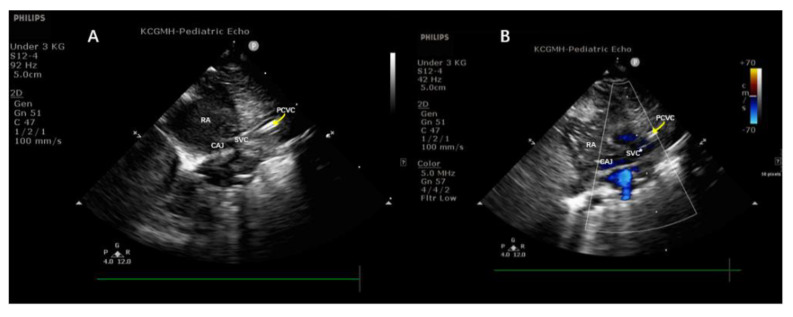
Normal saline is used to identify the tip location of PCVC. (**A**) RA is in a black color prior to usage of normal saline, and (**B**) while flushing with normal saline, the water jet shows in white, which extended from SVC to RA. CAJ, cavoatrial junction; PCVC, percutaneous central venous catheter; RA, right atrium; SVC, superior vena cava.

All PCVCs were inserted under sterile maneuvers and performed by the same nurse practitioner and neonatology fellow. The PCVCs used in the SVC group were 2.0 French single-lumen catheters, with internal stiffening stylet Bard Per-Q-Cath (Becton Dickinson, Covington, GA, USA). The ideal distance for insertion was estimated by the equations which were reported in our previous study [8]. The insertion of PCVC and the evaluation of the tip location were performed by a neonatologist fellow. The S12-4 sector transducer of the ultrasound device, Philips CX50 (Phillips Ultrasound, Bothell, WA, USA), was placed on this parasternal longitudinal view (Figure 1A), at the intersection of the left side of the baby’s sternum and the upper border between the nipples (Figure 1B) where the aorta was able to be seen clearly (Figure 1A). Then the probe was tilted slightly to the left side of the examiner, which is the right side of the patient, in order to position the SVC and right atrium (Figure 2B). In this view, the PCVC could be seen clearly in the SVC and near the cavoatrial junction (Figure 2A). The four-chamber view was also examined to ensure that the tip was not located inside the chamber. After the PCVC was clearly seen in the proper position, the stylet/guidewire would be removed from PCVC. To reconfirm the tip location, a minimal (less than 1 mL) volume of normal saline fluid was flushed via the catheter, which provided the dynamic of the flow and could be seen clearly on the ultrasonographic monitor (Figure 3, Supplementary Video S1). After ultrasonography, radiography was ordered for final examination and PCVCs could be repositioned if the tip was not near the cavoatrial junction, which was confirmed by another neonatologist who was blinded to the study. If the radiography was taken out of hours, the neonatologist could read the image via tablet and management would be taken by the duty resident doctor without any delay. As most PCVCs were prearranged insertion, thus the off-hour radiography exam was quite rare. The procedure in the IVC group was also performed as described previously [10]. The ultrasonography device and the material of PCVCs in the IVC group were similar to those in the SVC group, and the tips of PCVCs in IVC can be detected in the subcostal view. During the maintenance period, the dressing sterilization was based on the protocol of maximum sterile barrier precaution regular dressing changes every week, and when oozing occurred [13].

### 2.3. Statistical Analysis

To compare both groups, SVC and IVC, the data for gestational age, birth weight, gender, age, and body weight on the intervention date were examined. The duration of tip assessment by both ultrasonography and radiography, the procedure length of intervention, the repositioning rate, and the complication of PCVCs, such as CRBSI, occlusion, and unexpected removal were also analyzed for the comparison. Student’s *t*-test and Fisher’s exact test were used to analyzing continuous and categorical data, respectively. All tests were two-tailed and statistical significance was established as *p* < 0.05.

## 3. Results

Fifty PCVCs were placed in each SVC or IVC group during the study period. The mean gestational age in the groups of IVC and SVC was 31.43 ± 5.11 weeks (range: 26.28–36.18 weeks) and 32.16 ± 4.21 weeks (range: 29.43–35.11 weeks), respectively. The mean birth weight in the groups of IVC and SVC was 1642.18 ± 984.97 g (range: 820.00–2592.50 g) and 1792.00 ± 844.63 g (range: 1147.50–2387.50 g), respectively. The mean body weight at the moment of intervention in the groups of IVC and SVC was 2035.60 ± 1036.72 (range: 915.00–2430.00 g) and 1819.40 ± 1210.06 (range: 1243.75–2685.00 g). No significant differences were noted between the SVC and IVC groups in GA, BW, gender, age, and body weight at the moment of intervention, duration of intervention, and duration of radiography. The repositioning rate in the IVC group was 4% and in the SVC group was 16%, indicating no significant between-group difference (*p* = 0.09). Furthermore, in the SVC group, patients with/without invasive ventilation also did not have significant differences in all variables which were presented in Table 1. However, the mean duration of ultrasonography examination in the SVC group was 12.50 min, which was significantly longer than that in the IVC group (*p* < 0.001). Although the invasive ventilation group had a little longer duration than the non-invasive ventilation group, it was not significantly different. Besides, the complications of PCVCs, such as CRBSI, occlusion, and unexpected removal, were also insignificantly different between the two groups, as presented in Table 1.

## 4. Discussion

Results of the present study have demonstrated that the tip location of PCVCs inserted into the SVC can be detected accurately by ultrasonography. The rate of repositioning in the SVC was higher than that in the IVC group, but not significantly different. Thus, it becomes more critical to evaluate PCVC tips in the SVC by ultrasonography than those in the IVC. However, the mean time of the ultrasonography exam for PCVCs in the SVC was 12.5 min, which was significantly longer compared to locating PCVC tips in the IVC. However, ultrasonography was faster than radiography evaluation for PCVCs in either the SVC or IVC, which allows immediate administration of medications or nutritional supplementation with no delays. 

Although the repositioning rate of PCVC in SVC was higher than IVC group, it was not significantly different. The PCVC migration commonly happened within the first day [14]. Others had shown that the migration rate of PCVC in the upper extremity was higher than lower extremity but not significant, which was compatible with our result. Upper extremity PCVC could migrate to the jugular vein, which may cause serious complications [15]. Only one PCVC in the SVC group migrated to the jugular vein in this study and it was repositioned after the radiography exam. The risks of migration were associated with gender, difficult insertion of catheters, and dressing changes [14]. Serial follow-up of the tip location of PCVC could be taken whenever it is needed. The advantage of ultrasonography is its real-time assessment, portability, and lack of ionizing radiation, making it a convenient device in ICU.

The mean time for waiting for an X-ray examination was 150–160 min in this study which was a long time for an ICU order. The portable radiography in our hospital could be ordered urgently or not, but checking the location of PCVC is not urgent. Thus, it cannot be ordered at a definite time and must wait. However, there was no difference in time taken X-rays in and out of hours in our hospital because the number of portable radiography devices during the working hour was similar to during off-hours, which did not prolong the waiting time. 

Certain difficulties must be overcome during ultrasound assessment of PCVCs in the SVC. First, PCVC tips were hard to visualize in a collapsed SVC, which may occur as a result of positive pressure ventilation. Because increasing positive pressure of mechanical ventilation will decrease intrathoracic great venous return and reduce right ventricle preload, further blood flow is prevented [16]. However, the repositioning rate between patients with and without invasive ventilation was not different in this study, it might be due to the well-trained skill of ultrasonography for detecting the tip of PCVC. Secondly, arm movement is known to cause significant displacement on catheter tips in the SVC [17]. Catheters in different veins would typically migrate in a different direction with adduction or abduction of the arm. PCVCs in the basilic or axillary vein migrate toward the heart with adduction of the arm, whereas those via the cephalic vein do the opposite [18]. Besides, in our experience, extension or flexion of the forearm could also change the inside length of PCVCs. Thus, to prevent the tip from migrating further into the right atrium, monitoring the PCVC tip by real-time ultrasonography with the arm position at its maximal inward length is needed. The emphysematous lung is another difficulty during ultrasonography evaluation because the ultrasound waves are disturbed by air movement. Bronchopulmonary dysplasia is a common disease in premature neonates, which leads to air being trapped in the lungs. Thus, when a patient’s lung condition cannot be checked appropriately by ultrasonography, the best optimal PCVC puncture site would be on the lower limbs. Additionally, congenital heart diseases may present anomalous venous return. The most common congenital venous anomaly is a persistent left-sided SVC, which is seen in 0.3% to 0.5% of the general population [18]. The PCVC tip location may lie in the left SVC or within the coronary sinus, which is hard to be detected by ultrasonography. 

### Limitations

The present study has a few limitations, including that the two groups of different patients were enrolled at different times. Although all PCVCs were assessed by the same neonatology fellow, ultrasonography skills are expected to increase over time. The ultrasonography of PCVC in IVC had been performed for more than one year, which was longer than PCVC in SVC; besides, the IVC group data were reviewed retrospectively, and SVC group data were reviewed prospectively, which may cause certain biases. However, the repositioned PCVCs were not significantly increased in the SVC group in this study. Thus, the influence of the ultrasonography technique in this study was minimal. 

According to our experience, better ultrasonography skills may be needed in evaluating the SVC group than the IVC group, as so many obstacles must be overcome. Therefore, specialized training in the ultrasonography examination should be taken. Before the beginning of this study, the neonatologist fellow who performed the ultrasonography of PCVC in SVC had taken 30 cases as a training course to minimize the bias. 

## 5. Conclusions

In conclusion, ultrasonography provides an accurate and convenient method by which to assess PCVC tip location in the SVC and to help prevent serious complications that may result from malposition of PCVCs.

## Figures and Tables

**Table 1:** Demographic and complication data between the inferior vena cava (IVC) and superior vena cava (SVC) groups.

	IVC (*n* = 50)	SVC (*n* = 50)				*p*-Value
		Invasive Ventilation (*n* = 13)	Non-Invasive Ventilation (*n* = 37)	*p*-Value	Total	(IVC vs. SVC)
Gestational age (wk)	31.43 ± 5.11	30.87 ± 5.10	32.63 ± 3.83	0.27	32.16 ± 4.21	0.43
BW (g)	1642.18 ± 984.97	1725.77 ± 847.87	1815.27 ±853.96	0.75	1792.00 ± 844.63	0.42
Gender (M/F)	33/17	9/4	26/11	1.00	35/15	0.41
Age at the moment of intervention (days)	14.60 ± 23.90	25.46 ± 29.86	17.54 ± 26.74	0.41	19.60 ± 27.49	0.33
BW at the moment of intervention (g)	2035.60 ± 1036.72	2092.85 ± 898.87	2015.49 ± 1091.78	0.80	1819.40 ± 1210.06	0.34
Duration of intervention time (min)	10.13 ± 8.07	14.38 ± 10.29	12.49 ±11.34	0.58	12.98 ± 11.00	0.15
Duration of echo examination (min)	3.17 ± 1.72	13.62 ± 11.10	12.11 ± 8.02	0.60	12.50 ± 8.82	< 0.001
Duration of X-ray examination (min)	149.32 ± 115.30	165.46 ± 71.06	159.10 ± 87.79	0.80	160.80 ± 83.10	0.57
Repositioning rate (%)	4.00%	0	21.62%	0.09	16.00%	0.09
Unexpected removal	20.00%	30.77%	29.73%	1.00	30.00%	0.20
CRBSI (‰)	3.20‰	0	6.90‰	0.19	4.77‰	0.53
Occlusion (%)	14.00%	7.69%	13.51%	1.00	12.00%	1.00

The data were presented as mean ± standard deviation. Student’s *t*-test was used for continuous variables. Fisher’s exact test was used for categorical variables. BW, body weight; CRBSI, catheter-related bloodstream infection IVC, inferior vena cava; M/F, male/female; SVC, superior vena cava.

## Data Availability

The data presented in this study are available on request from the corresponding author. The availability of the data is restricted to investigators based in academic institutions.

## Supplementary Materials

The following supporting information can be downloaded at: https://www.mdpi.com/article/10.3390/children9050624/s1, Video S1: Dynamic flow on the monitor during normal saline flushed.
